# Resection of intracardiac leiomyoma originating from the inferior vena cava through a single median sternotomy incision using a silk suture snare technique: a case report

**DOI:** 10.1186/s12872-023-03630-z

**Published:** 2023-11-30

**Authors:** Ting Xie, Matiullah Masroor, Cong Liu, Shengxiong Lin, Jing Song, Zhengping Wang, Xuan Chen

**Affiliations:** 1 Department of Cardiac Surgery, Hainan General Hospital (Hainan Affiliated Hospital of Hainan Medical University, Haikou, 570311 China; 2 Department of Cardiovascular Surgery, Union Hospital, Tongji Medical College, Huazhong University of Science and Technology, Wuhan, 430022 China; 3 Department of Cardiothoracic and Vascular Surgery, Amiri Medical Complex, Qargha Road, Kabul, Afghanistan; 4 Department of Ultrasound Medicine, Union Hospital, Tongji Medical College, Huazhong University of Science and Technology, Wuhan, 430022 China; 5 Department of Cardiothoracic Surgery, Ezhou Central Hospital, Ezhou, 436000 China; 6 Department of Radiology, Hainan Traditional Chinese Medicine Hospital, Hainan, Haikou, 570203 China; 7 International College of Nursing, Hainan Vocational University of Science and Technology, Haikou, 570216 China

**Keywords:** Cardiac tumor, Leiomyoma, Sternotomy, Inferior vena cava, Complete resection

## Abstract

**Background:**

Intracardiac leiomyoma is a rare benign right heart tumor that usually extends from the intravenous system. The patient often has a history of uterine leiomyoma.

**Case presentation:**

We report a 46-year-old female patient who presented to us with exertional dyspnea, chest tightness, and shortness of breath for two weeks and had a history of uterine leiomyoma resection. Echocardiography showed a pedunculated solid mass in the right heart with the pedicle attached to the inferior vena cava. The surgery was performed under cardiopulmonary bypass established through the femoral artery and vein with a probable diagnosis of leiomyoma. The tumor was removed by ingenious surgical technique: a snare made of silk suture in which the tumor’s pedicle was trapped, and the tumor with its pedicle was carefully removed with the help of a scalpel along the silk suture. The histopathology report confirmed the diagnosis of intravenous leiomyoma. The postoperative course was uneventful and the patient was discharged a week later.

**Conclusion:**

Intracardiac leiomyoma is a rare benign smooth muscle tumor. Surgery is the mainstay of treatment with different surgical approaches available. It is possible to completely remove cardiac leiomyomas through sternotomy without the need for an abdominal incision if the leiomyoma is originated in the inferior vena cava not far from the right atrium.

## Background

Intravenous leiomyomatosis (IVL) is a rare and special type of uterine leiomyoma encountered in clinical practice in which leiomyomatous tissue is formed within the uterine vessels and progresses through the vessels. The median age of occurrence is 44 years and patients present usually with abnormal uterine bleeding or abdominal mass etc. [1]. IVL develops only in 0.1% of the patients with uterine leiomyoma [1, 2]. IVL can invade and metastasize the iliac veins, inferior vena cava (IVC), and even the heart [2]. IVL which invades the heart is referred to as intracardiac leiomyoma (ICL) which is even rare. The cardiac involvement of the IVL is 10–40% [1, 3]. ICL is a benign smooth muscle tumor of extracardiac origin histologically, invading or metastasizing the heart through the veinous system [4]. ICL patients often have a history of uterine leiomyoma, hysterectomy, or myomectomy [4]. It is usually diagnosed in the fifth decade of life [4]. IVL usually extends to the heart from the venous system but isolated cases arising from the heart wall have been reported [2, 3]. ICL can be fatal due to progression to heart failure and total outflow obstruction if left untreated. Echocardiography combined with computed tomography (CT) or magnetic resonance imaging (MRI) can help in diagnosis but definitive diagnosis is made by histopathology. Generally, after diagnosis, the surgery is performed through a combined thoracoabdominal incision or abdominal incision for the IVC or its branches for embolectomy [3]. In this paper we report a case of complete removal of ICL arising from the IVC close to the right atrium through a median sternotomy without an abdominal incision using a silk suture snare technique.

## Case presentation

**Fig. 1:**
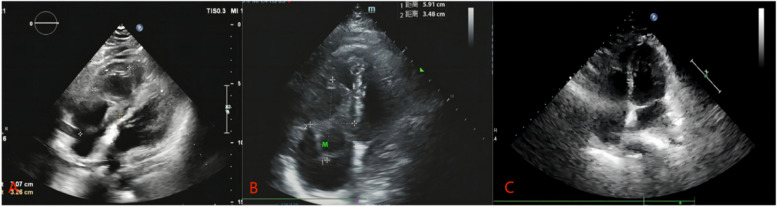
**A-B** Preoperative echocardiography images show the presence of the tumor. **C** Postoperative echocardiography image shows normal heart chambers without tumor

**Fig. 2:**
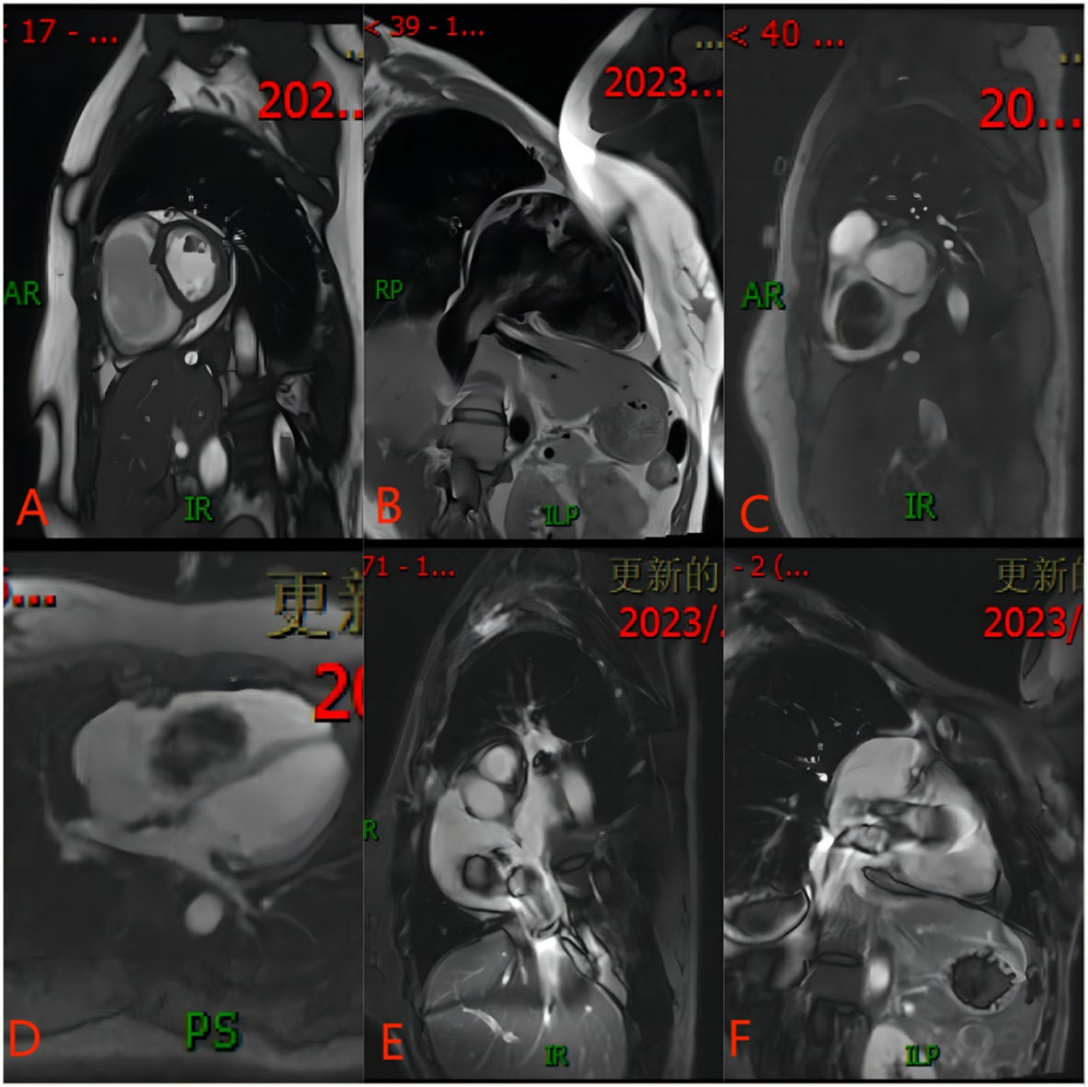
**A-C** shows the short axis view of the ventricle, atrium, and inferior vena cava opening of MR cardiac cine bright blood sequence, the tumor in the right atrium and inferior vena cava is low signal; **D** shows the four chamber bright blood sequence, shows that the tumor cross the tricuspid valve and enter the right ventricle; **E**, **F** shows the two-chamber view of cardiac cine bright blood sequence and T2 sequence passing through the inferior vena cava, the tumor extends from the hepatic segment of inferior vena cava to the right ventricle

**Fig. 3:**
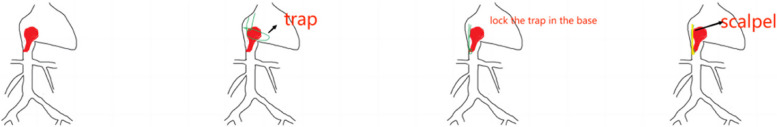
Schematic diagram of surgical resection of tumor

**Fig. 4:**
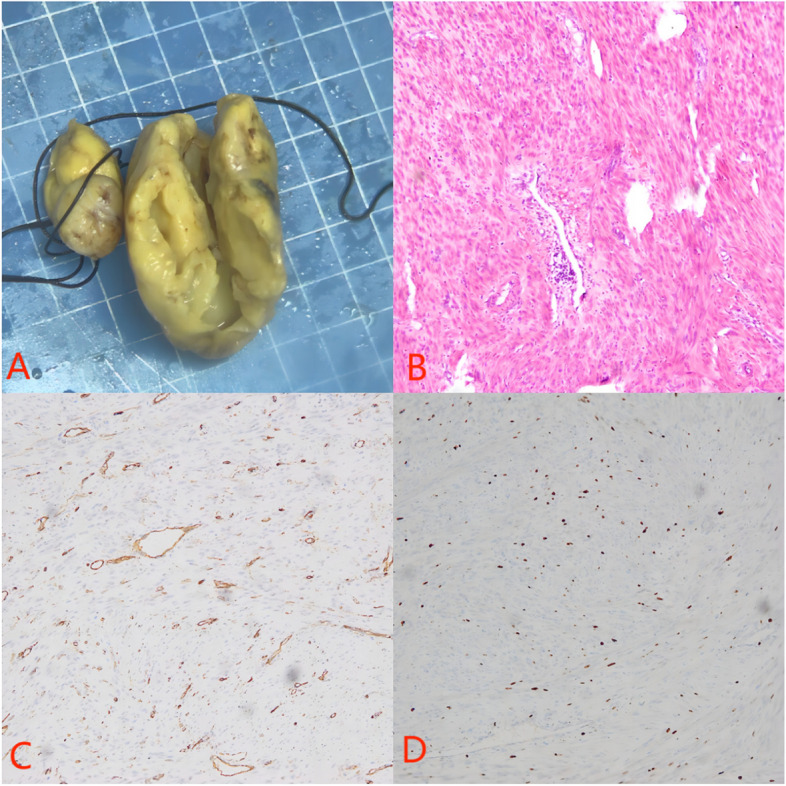
**A** Gross specimen **B**. HE staining(× 100) **C**. Immunohistochemical staining CD34 -ve **D**. Immunohistochemical staining SMA + ve

A 46-year-old female patient presented to our hospital with exertional dyspnea, chest tightness, and shortness of breath for two weeks. She had a history of uterine myomectomy four years ago. On physical examination, her blood pressure was 116/70 mmHg, pulse 78 beats/minute, and RR was 20/minute. Inspection of the chest revealed no abnormal findings. There was no abnormality found on the auscultation of the chest as well. Routine blood and biochemical tests were normal. Echocardiography showed a pedunculated solid hypoechoic mass in the right heart with the pedicle attached to the inferior vena cava not far from its opening into the right atrium (Fig. 1). In combination with the past medical history, angioleiomyoma was considered. Lower limb Doppler ultrasound showed plaques in bilateral dorsalis pedis arteries. A gynecological ultrasound showed an isoechoic mass on the right side of the uterus (possible broad ligament fibroid) with small uterine leiomyoma. CT showed that the right atrium had a slightly low-density focus with a size of 3.7*2.2 cm. Magnetic resonance imaging (MRI) showed that leiomyoma originated from the inferior vena cava (approximately 3 cm from the opening of the inferior vena cava into RA) and occupies the right atrium (Fig. 2). Open cardiac surgery through median sternotomy was performed under general anesthesia with a cardiopulmonary bypass established with right femoral artery and vein and an additional venous cannula in the superior vena cava. The tumor was completely resected with tricuspid valve plasty and temporary pacemaker placement. During surgery, a solid mass of about 6*4 cm in size was located in the right atrium with its pedicular attachment in the posterior wall of the inferior vena cava about 3 cm away from its opening into the atrium. The capsule of the mass was complete. The leaflets of the tricuspid valve were normal with annular enlargement which was causing incomplete closure of the valve. In this case, because the pedicel of cardiac leiomyoma originated close to the opening of the inferior vena cava into the right atrium, after the peripheral establishment of cardiopulmonary bypass the silk suture was used to make a snare to cover and ligate the pedicel of the tumor. A small scalpel was then carefully slide down the silk suture to remove the tumor with its pedicle, avoiding abdominal incision (Fig. 3). The histopathology report showed CD34 negative and smooth muscle actin (SMA) positive markers suggesting vascular leiomyoma (Fig. 4). The recovery was uneventful and the patient was discharged one week later.

## Discussion

Intravenous leiomyomatosis is often seen in perimenopausal women, which is clinically rare and often associated with a history of uterine leiomyomas. It can grow along the vasculature in a very aggressive fashion. Uterine venous leiomyomas involving the right heart are extremely rare and available literature is mostly individual cases [5, 6].

IVL confined to the inferior vena cava has no obvious symptoms, but at the time symptoms occur, most tumors have invaded the heart. There is lack of effective standards for etiology, classification, and treatment methods. Regarding the origin of the ICL, one view is that the tumor originates from the venous vascular wall in the uterus or pelvis. Other holds the view that the ICL is a further extension of the uterine leiomyoma to the vasculature [7]. Most ICLs extend from the uterus to the heart but interestingly, although some patients have a history of uterine leiomyoma but uterine leiomyoma and cardiac leiomyoma do not have anatomical continuity and ICL arises from the heart walls itself [2]. Due to its extension from pelvis to the heart, IVL is considered a benign tumor with malignant behavior [3]. In this case, there was a history of uterine leiomyomectomy, however, the pedicle of cardiac leiomyoma originated from the inferior vena cava. Li et al. [4] found that ICL originating from the uterus usually has a serpentine appearance macroscopically, with IVL extends from ovarian or iliac vein into the IVC and RA. In contrast, some cases with ICL arising from the venous smooth muscle wall itself have been reported [4].

The tumor is generally smooth and rubbery, with a greyish-white or rusty color, with or without an intumescent intracardiac head. In patients with a long course of this disease, the tumor may be calcified and extended more than 50 cm [4]. Immunohistochemical staining is positive for SMA, a smooth muscle markers [8]. Evidence suggests that the etiology of cardiac leiomyoma is not only related to uterine leiomyomatosis but may also be related to other factors. Current literature reports that the vast majority of ICLs occur in perimenopausal women [4], which suggests that the tumor is closely related to the estrogen changes in the body. Generally, echocardiography is a more convenient method and can provide real-time information about an intracardiac tumor. CT and MRI can clarify the tumor anatomy and its relation with adjacent structures.

In terms of ICL morphological characteristics, to achieve the goal of complete tumor resection, the general surgical procedure needs to involve the pelvic cavity, abdominal cavity, thoracic cavity, and great vessels. It requires multidisciplinary team cooperation involving cardiovascular surgeon, general surgeon, and gynecology and obstetrics surgeon. Generally, a combined thoracoabdominal incision under extracorporeal circulation is used to remove the tumor [3, 9, 10]. In this case, according to the location of the pedicel of cardiac leiomyoma which originated about 3 cm from the inferior vena cava opening into the RA, a single median sternotomy was done. After the establishment of CPB, the silk suture was used to make a snare to cover and ligate the pedicel of the tumor, and then a scalpel was used to remove the tumor, avoiding an abdominal incision. This approach theoretically improves the feasibility of successful single sternotomy incision surgery and eliminating the need for an abdominal incision even if the pedicle of the tumor is far but within a reachable location from the opening of IVC. A single abdominal incision approach with peripheral CPB has been performed for ICL without intracardiac portion dilation of the tumor [6]. One-stage and two-stage operation for ICL has also been reported [3, 7, 11]. An individualized and reasonable surgical plan is required on the premise of fully assessing the patient history, imaging data, and surgical tolerance. Research shows that complete resection has good short-term and long-term results, with no reports of recurrence or postoperative death [4, 12]. However, in 33.3% of patients, recurrence occurs after incomplete resection [4]. Recurrence of ICL is not associated with favorable outcomes [5]. Therefore, close postoperative follow-up is necessary.

Combined with medical history, echocardiography, CT, and other tools can help in diagnosis, and diagnosis is generally not difficult. Once ICL is diagnosed, curative care should be carried out, and the surgical plan should be fully evaluated as far as possible. Individual incisions should be formulated according to the scope and origin of the tumor, and multiple and large incisions should be avoided as far as possible. Follow-up and observation should be carried out to detect tumor recurrence at an early stage.

## Conclusion

Once ICL is diagnosed, it should be actively treated with complete surgical resection, if possible, which has a good prognosis. The tumor usually is widely invading and growing. The surgical plan should be formulated under a multidisciplinary team and should be fully evaluated and individualized as far as possible. Close follow-up and observation are required to improve the survival of patients and prevent tumor recurrence.

## Data Availability

All data generated and analyzed during this study are included in this published article.

## References

[1] Du J, Zhao X, Guo D, Li H, Sun B (2011). Intravenous leiomyomatosis of the uterus: a clinicopathologic study of 18 cases, with emphasis on early diagnosis and appropriate treatment strategies. Hum Pathol.

[2] Yano M, Katoh T, Nakajima Y, Iwanaga S, Kin R, Kozawa E, Yasuda M (2020). Uterine intravenous leiomyomatosis with an isolated large metastasis to the right atrium: a case report. Diagn Pathol.

[3] Chiang CS, Chen PL, Kuo TT, Chen IM, Wu NY, Chang HH (2018). One-stage surgery for removal of intravascular leiomyomatosis extending to right ventricle. Medicine (Baltimore).

[4] Li B, Chen X, Chu YD, Li RY, Li WD, Ni YM (2013). Intracardiac leiomyomatosis: a comprehensive analysis of 194 cases. Interact Cardiovasc Thorac Surg.

[5] Gan HL, Zhang JQ, Zhou QW, Kong QY, Zhao S, Bo P (2011). Surgical treatment of intracardiac leiomyomatosis. J Thorac Cardiovasc Surg.

[6] Li H, Xu D, Lu W, Wang C (2016). Complete resection of intracardiac leiomyomatosis through an abdominal approach under peripheral cardiopulmonary bypass. J Thorac Cardiovasc Surg.

[7] Worley MJ, Aelion A, Caputo TA, Kent KC, Salemi A, Krieger KH, Goldstein MJ, Kuo DY, Slomovitz BM (2009). Intravenous leiomyomatosis with intracardiac extension: a single-institution experience. Am J Obstet Gynecol.

[8] Ricci MA, Cloutier LM, Mount S, Welander C, Leavitt BJ (1995). Intravenous leiomyomatosis with intracardiac extension. Cardiovasc Surg.

[9] Iacona GM, Harb S, Krishnamurthi V, Yun JJ (2023). Intracaval Leiomyoma with Intracardiac Extension. Int J Angiol.

[10] Li H, Xu J, Lin Q, Zhang Y, Zhao Y, Tong H, Tu R, Xu D, Wang C, Lu W (2020). Surgical treatment strategies for extra-pelvic intravenous leiomyomatosis. Orphanet J Rare Dis.

[11] Castelli P, Caronno R, Piffaretti G, Tozzi M (2006). Intravenous uterine leiomyomatosis with right heart extension: successful two-stage surgical removal. Ann Vasc Surg.

[12] Anselmi A, Tsiopoulos V, Perri G, Palladino M, Ferrante A, Glieca F (2010). Case series of resection of pelvic leiomyoma extending into the right heart: surgical safeguards and clinical follow-up. J Cardiovasc Med (Hagerstown).

